# Psychosocial issues and coping strategies in families affected by long‐chain fatty acid oxidation disorders

**DOI:** 10.1002/jmd2.12402

**Published:** 2023-12-05

**Authors:** Maren Thiel, Sven F. Garbade, Stefanie Rosenbaum‐Fabian, Ute Spiekerkoetter, Sarah C. Grünert

**Affiliations:** ^1^ Department of General Pediatrics, Adolescent Medicine and Neonatology Faculty of Medicine, University Medical Center, University of Freiburg Freiburg Germany; ^2^ Division of Pediatric Neurology and Metabolic Medicine, Center for Pediatric and Adolescent Medicine University Hospital Heidelberg Heidelberg Germany

**Keywords:** coping, disease burden, fatty acid oxidation defects, fatty acid oxidation disorders, quality of life

## Abstract

Long‐chain fatty acid oxidation disorders (lcFAODs) are associated with a high disease burden due to both the risk of metabolic decompensation as well as chronic, partly irreversible complications in some. Little research has been performed on the impact of these disorders on the daily life of parents and caregivers. We performed a web‐based questionnaire study among parents/caregivers of patients affected with lcFAODs. The questionnaire focused on challenges at different ages of the child, on disease management issues, schooling, family and social life as well as the parental job situation, and their overall attitude toward the disease and the future life of their child. Data were collected from parents/caregivers of 63 patients (87 respondents, 63% mothers, 36% fathers) with lcFAODs (median age of patients 8.0 years, range 0–25 years, long‐chain 3‐hydrocyacyl‐CoA dehydrogenase deficiency 40%, mitochondrial trifunctional protein deficiency 14%, very long‐chain acyl‐CoA dehydrogenase deficiency 41%, carnitine palmitoyltransferase 2 deficiency 5%). The overall disease burden of parents was considered highest during infancy and decreased with increasing age of their child. More than one third of parents were afraid that their child's disease might have an impact on his/her career choice and adult life. Negative effects of the child's disease on the job situation and career development were more commonly reported by mothers compared to fathers. Although the majority of parents considered their child's metabolic disorder a severe disease, most parents had a positive attitude toward their child's disease and seemed to cope well with their situation.

**Synopsis**

Although most parents/caregivers of patients with a long‐chain fatty acid oxidation disorder (lcFAOD) consider their child's metabolic disorder a severe disease, the majority thinks that life with a lcFAOD is well manageable, has a positive attitude toward the disease, and seems to cope well with their situation.

## 1 BACKGROUND

Long‐chain fatty acid oxidation disorders (lcFAODs) comprise several rare inborn errors of metabolism that affect either the transport of long‐chain fatty acids into the mitochondria via the carnitine shuttle or the mitochondrial betaoxidation of long‐chain fatty acids. LcFAODs are clinically characterized by fasting intolerance with hypoketotic hypoglycemia, muscular symptoms including muscle weakness and episodic rhabdomyolysis as well as possible cardiomyopathy.^1^ Affected patients are prone to metabolic decompensation which is usually triggered by catabolism. Individuals with defects of the mitochondrial trifunctional protein (MTP) complex (long‐chain 3‐hydrocyacyl‐CoA dehydrogenase [LCHAD] deficiency OMIM #609016, and MTP deficiency #609015) may additionally present with peripheral neuropathy and retinopathy.^2^, ^3^

The mainstay of therapy at least in severe forms is a fat‐restricted diet supplemented with medium‐chain triglycerides, and the prevention of catabolism by frequent meals. Especially, young patients may require nocturnal gastric tube feeding or nighttime meals. While most muscular symptoms are amenable to dietary treatment, retinopathy and neuropathy may develop despite optimal dietetic compliance.^2^ Patients with lcFAODs reach adulthood and some have rather minor symptoms during the course of the disease until later age. If the overall life expectancy in lcFAODs is affected is still unknown.

Medical conditions, particularly those requiring strict adherence to treatment recommendations or dietary restrictions, can be associated with impaired quality of life (QoL) and emotional functioning.^4^, ^5^, ^6^, ^7^, ^8^, ^9^, ^10^ Patients and caregivers do not only experience a loss of spontaneity in their daily life, but also have to live with the risk of potentially life‐threatening emergency situations and, in the case of LCHAD and MTP deficiencies, possible irreversible long‐term complications.

Only few studies have focused on the QoL of patients and caregivers with metabolic disorders, and especially, studies on patients with lcFAODs are scarce. Williams‐Hall et al. have recently investigated the QoL impacts associated with lcFAODs via a focus group and semi‐structured interviews with a small number of patients, caregivers, and expert clinicians.^11^ In this study, lcFAODs were reported to have a significant impact on various aspects of patients' lives including physical functioning, participation in daily activities, emotional/psychological well‐being, and social functioning.^11^ The aims of this study were to (1) describe the challenges of parents and caregivers to care for a child with a lcFAOD and the impact of the disease on different areas of life including the development of coping strategies; (2) explore the differences between mothers and fathers with respect to the different areas of professional, private and family life; and (3) generate an understanding of the unmet needs to further improve the management of lcFAOD.

## 2 METHODS

A questionnaire was designed to assess the psychosocial burden of parents/caregivers with a child affected by a lcFAOD. Doctors, dieticians, caregivers, patients, and psychologists were involved in the development of the survey. The questionnaire addressed the diagnostic process, the clinical course and challenges of different age groups, dietary management, siblings, job situation, social life, sports, traveling, as well as the parents'/caregivers' attitude toward their child's disease. The aim of this explorative study was to elucidate how disease‐specific facets of lcFAODs interfere with normal adult life of caregivers. Therefore, we decided against using a standardized generic questionnaire, but, in contrast, focused on disease‐specific questions to assess how parents/caregivers struggle and cope with the medical condition of their children in daily life. The questionnaire was not validated with another psychological instrument. An English translation of the questionnaire can be found in the Supplemental Material. The study was performed as an online survey using a RedCap platform. Caregivers of children with lcFAODs were recruited via the patient organization for fatty acid oxidation disorders in Germany, Fett‐SOS e.V., and the metabolic center of the University Children's Hospital Freiburg, Germany. As the study also aimed to investigate possible differences between mothers and fathers, both parents/caregivers were asked to fill in the questionnaire independently for their child/children. The study was approved by the ethics committee of the university hospital Freiburg (EKFR Nr. 20‐1132). All participants gave their informed consent. The online survey was open from December 2020 to March 2021.

### 2.1 Statistical analysis

Data analysis was performed using the Software R (https://www.r-project.org).^12^ Descriptive and explorative analysis was used to describe the study sample. Continuous data is reported with mean and standard deviation, count data is presented as frequencies and percentages. No a priori hypotheses are tested.

## 3 RESULTS

### 3.1 Study cohort

A number of 119 questionnaires were registered. Questionnaires without pseudonymization (*n* = 15), eight incomplete questionnaires for which a second complete questionnaire was available, one questionnaire filled in by a dietician, and two questionnaires with only master data were excluded from the analysis. The remaining 93 questionnaires were answered by a total of 87 parents/caregivers (63% mothers, 36% fathers, and 1% foster parents). These 93 questionnaires provide information on 63 patients with lcFAODs (52% female and 48% male). The age of the patients ranged from 0 to 25 years with a median age of 8.0 years. Forty‐one percent of patients had very long‐chain acyl‐CoA dehydrogenase (VLCAD) deficiency, 54% LCHAD or MTP deficiency, and 5% carnitine palmitoyltransferase 2 (CPT2) deficiency. For 30 patients, both mother and father filled in the questionnaire. Ninety‐one percent of the respondents were German, and 7% were Austrian.

Eighty‐six percent of patients (*n* = 54) were detected by newborn screening (NBS), thereof 74% before the onset of first symptoms. Seven patients (11%) were diagnosed symptomatically either in the prescreening area or after a false‐negative NBS result, while two children (3%) were identified without clinical symptoms via family screening due to an older affected sibling.

### 3.2 Initial diagnosis

In only 58% of cases, the initial diagnosis was communicated to the parents by a metabolic physician. In one third, the conversation was conducted by a physician without metabolic expertise. In only 13% of the cases, a dietician was involved, and in only 1% a psychologist was present. In particular, the lack of expertise of the physician about the disease, the expected clinical course, and possible therapy made parents feel insecure during the initial consultation. On the other hand, some parents reported to have been overwhelmed by too much expert information. As a result, the majority of parents rated the diagnostic opening as rather bad (mean rating 4.26 if information was provided by a metabolic expert versus 2.96 in cases in which information was received from a physician not experienced in metabolic diseases; scale 1–7 with 1 = very bad, 7 = very good). The three most commonly reported emotional reactions of caregivers following the diagnosis were fear (79%), concern (79%), and sadness (64%). Sixty‐four percent of caregivers felt overwhelmed during the diagnostic process, and 25% reported to have been discouraged. Psychological support was only offered in 20% of cases.

### 3.3 Challenges of the diet

Ninety‐three percent of mothers and 57% of fathers reported being responsible for or involved in the diet of their child/children and the preparation of meals. During infancy, 87% of patients only had oral feeds, while 6% required partial tube feeding, and 5% were fully tube‐fed. Between the age of 1–6 years, 19% required tube feeding (7% partial tube feeding, 12% fully tube‐fed). For 18 parents, tube feeding led to a relaxation of the feeding situation, while 8 parents experienced the nasogastric/percutaneous endoscopic gastrostomy (PEG) tube as an additional burden.

Patients usually had between one to six nighttime meals (mean 1.9 meals/night). These were organized very differently by the families. Most parents shared the responsibility for nighttime meals (alternating feeds during the night, alternating days); however, mothers carried the main load. In 8 of 10 cases, in which only one parent was responsible for the nighttime meals, the mother was the responsible person. Fifty‐seven percent of mothers and 49% of fathers reported sleeping problems due to interrupted nights' sleep (rank of ≥4 on a scale from 1 to 7), and 63% and 57% of mothers and fathers, respectively, felt not fully efficient during the day (rank of ≥4 on a scale from 1 to 7) due to night sleep disruption.

Eating in a restaurant may be challenging for patients that require a special diet. Caregivers reported that 23% of patients (14/61) only ate self‐prepared food. Fifty‐one percent of children (31/61) regularly ate at kindergarten or school, 59% (36/61) at their friend's house, and 74% (45/61) with relatives. However, especially in kindergarten and school, parents/caregivers often prepared and provided the meals for their children.

Twenty‐four percent of caregivers reported that they had never eaten at a restaurant with their child. However, the majority considered eating out as nonproblematic, since options according to the dietary requirements are usually available.

**FIGURE 1:**
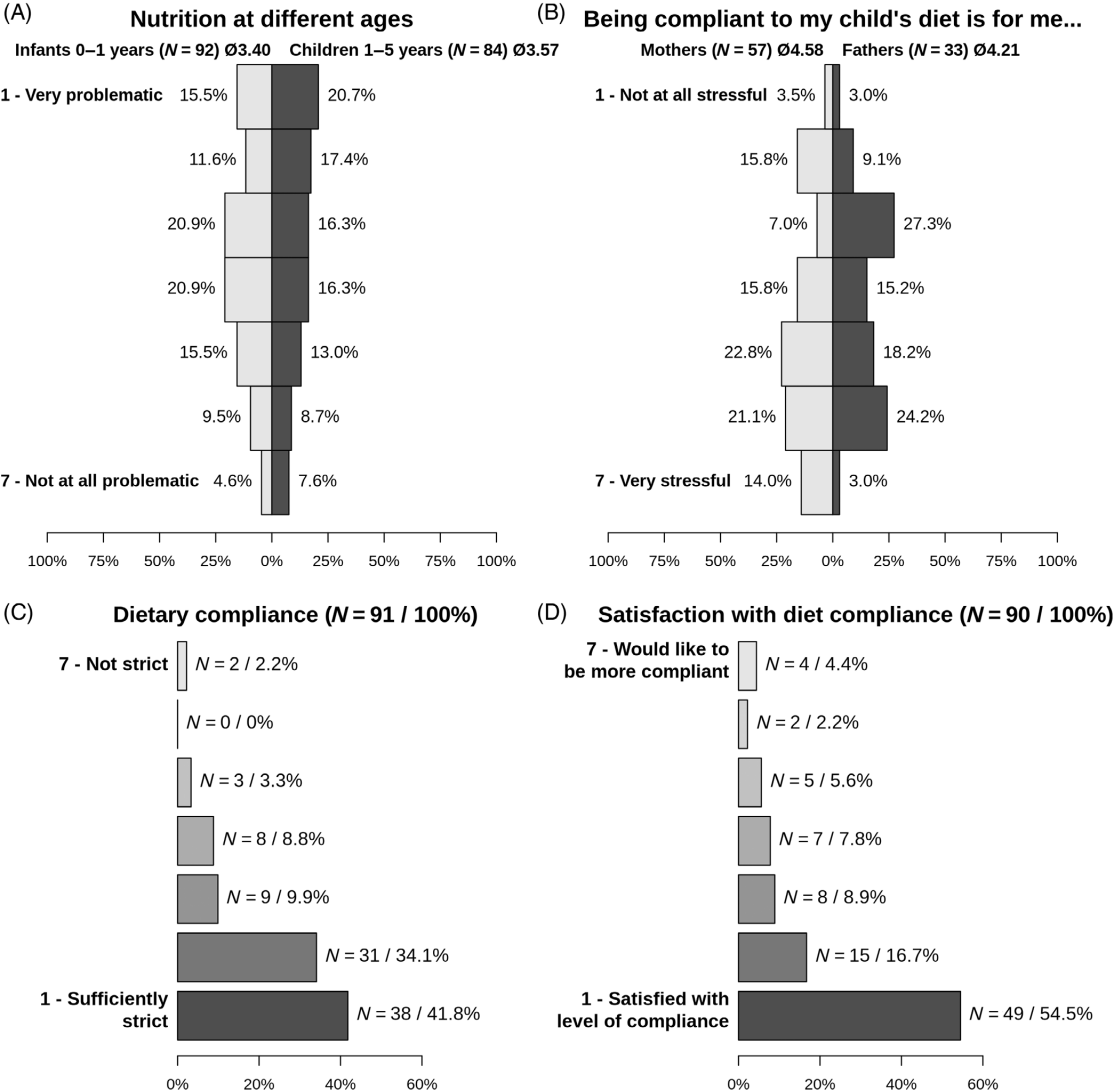
Perceived parental stress related to the diet of a child with lcFAOD. (A) Perceived parental burden related to the diet of the child at different ages. (B) Parental stress related to compliance with the diet. (C) Strictness of the diet. (D) Parental satisfaction with the own compliance with the child's diet. lcFAOD, long‐chain fatty acid oxidation disorder.

Caregivers were asked to rank the challenges of their child's diet during infancy and toddlerhood on a scale from 1 (very challenging) to 7 (not at all challenging) (Figure 1A). Figure 1A shows that the caregiver's views were highly variable. Overall, caregivers tended to regard their child's diet as less challenging with increasing age (in average 3.40 at age 0–1 year vs. 3.57 at age 1–5 years). Mothers tended to feel more stressed by controlling their children's diet than fathers (Figure 1B). The majority of parents considered themselves as consequent with regard to dietetic adherence: 76% feel sufficiently strict and 71% are satisfied with their level of compliance (ranks 1–2 on a scale from 1 to 7) (Figure 1C).

### 3.4 Management of emergency situations and hospital admissions

**FIGURE 2:**
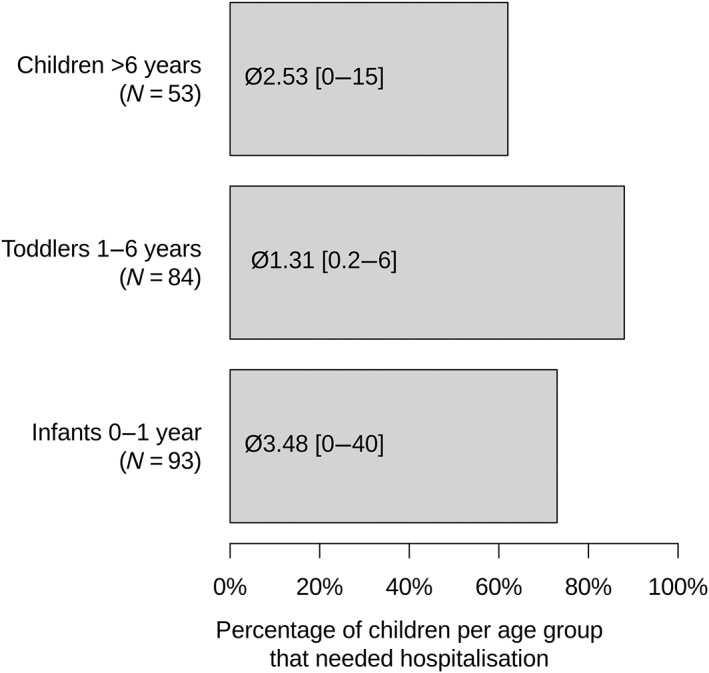
Need for hospitalization per age group (in % of patients in this age group) and frequency of hospitalizations per year (mean and range).

More than 90% of caregivers reported to often handle mild infectious diseases at home (92% ranks 5–7 on a scale from 1 to 7). Only 84% of patients had an “unwell” or emergency plan for such situations. An emergency document for hospital admissions was available in 92% of patients. The number of hospital admissions in different age groups is shown in Figure 2.

Seven patients had an implanted port catheter system. Eighty percent of patients were vaccinated according to the national recommendations, 18% only had selected vaccinations, and one patient was not vaccinated at all. Usually, no special precautionary measures were taken for routine vaccinations (71%). In 9% of children, the first vaccination was given in an in‐patient setting, and 22% of patients received prophylactic antipyretic drugs. In 12%, the diet was adjusted for 1–2 days according to the patients' “unwell plan.”

### 3.5 Clinical management and long‐term monitoring

Almost all patients were regularly followed in a metabolic center. Approximately, one‐fifth of caregivers expressed that they did not feel well informed about medical findings and results of follow‐up investigations. Parents/caregivers reported that 58% of the patients/children were afraid of the routine consultations in the clinic, at least during a certain age period, while 42% had no fear of routine follow‐ups. As expected, patients were especially afraid of the blood draws. As dietary management is the mainstay of treatment for lcFAODs, most caregivers (86%) are in close contact with a dietician which they can contact if there are any dietary questions. One third of families already consulted another metabolic center for a second opinion.

### 3.6 Challenges at different ages and parental stress

Challenges of living with a chronic disease may change dependent on the age of the patients as different developmental tasks have to be accomplished.

During infancy, 14% of patients required special therapies such as physiotherapy (eight patients), speech therapy (six patients), occupational therapy (four patients), and oral therapy (one patient). When asked for the greatest burden during infancy, parents/caregivers mentioned, among others, a fixed feeding schedule with short fasting times, feeding by the clock without hunger, lack of sleep, small amounts of drinking, fear of energy deficiency, and metabolic crises, insecurity and uncertainty about the child's development and long‐term complications. Positive experiences at this age included: age‐appropriate development of the child with the feeling that the diet was manageable, happy and satisfied baby, and support from family and friends.

During early childhood (age 1–3 years), 31% of patients were cared for at home, and 47% percent of patients had normal kindergarten/daycare, while 22% received special care (i.e., integrative kindergarten). More than 50% of parents reported that they were afraid of their child being admitted to out‐of‐home care. While the majority of parents/caregivers did not report any social problems in the kindergarten, few parents/caregivers said that their child was teased (*n* = 4) or experienced exclusion (*n* = 7). Thirty‐seven percent of parents/caregivers reported that their child also had positive experiences due to his/her disease. Thirty‐eight percent of patients in this age group received special therapy (physiotherapy, speech therapy, occupational therapy, etc.). Among the most challenging things during early childhood, parents/caregivers mentioned the eating rhythm and continued need for night meals, adherence to the diet and explanation of it to the child, fear of metabolic decompensations and hospitalizations in case of infections, and search for the diagnosis in cases of false‐negative screening or patients born in the prescreening era. Positive experiences included the increasing understanding regarding diet and communication of own needs on the part of the child, settling into a certain routine, normal development of the child despite inpatient stays, acceptance of the disease by kindergarten friends, support by family, friends, daycare center, and employer.

At school age (6–16 years, *n* = 36), 64% of patients went to a normal elementary school (22% of them with integration status and 13% with an individual case assistant), while 11% received special schooling. Thirty‐one percent attended a middle school or comprehensive school and 36% attended a high school. Some students experienced many absences from school due to their disease: more than 30 days per year in 8% of patients, 11–20 days per year in 19% of patients, less than 10 days/year in 42% of patients, and no sick leave in 28% of patients. In addition, nearly 90% of school‐age children had school absences due to routine check‐up appointments with specialists or the metabolic outpatient clinic. Students usually communicated their disease openly with classmates; however, 15% of primary school and 10% secondary school students, respectively, did not want their classmates to know about their disease. Eighteen percent and 14% of students felt excluded because of their metabolic disorder, and almost half of all students (42% and 48% of primary and secondary school students, respectively) could not participate in certain school activities. When asked for the greatest burden during school age, parents/caregivers mentioned, among others: school trips and increasing autonomy or beginning of puberty with nonadherence to diet, metabolic crises, lack of understanding and support from state institutions and teachers, physical complaints and secondary diseases due to the metabolic disorder, not being able to participate in or being excluded from school activities. Positive experiences from parents' view at this age included: the increasing independence and understanding of the child about his/her metabolic disorder and diet, accompanied by a more stable metabolic condition and positive development of the child, and acceptance and support from friends and teaching staff.

**FIGURE 3:**
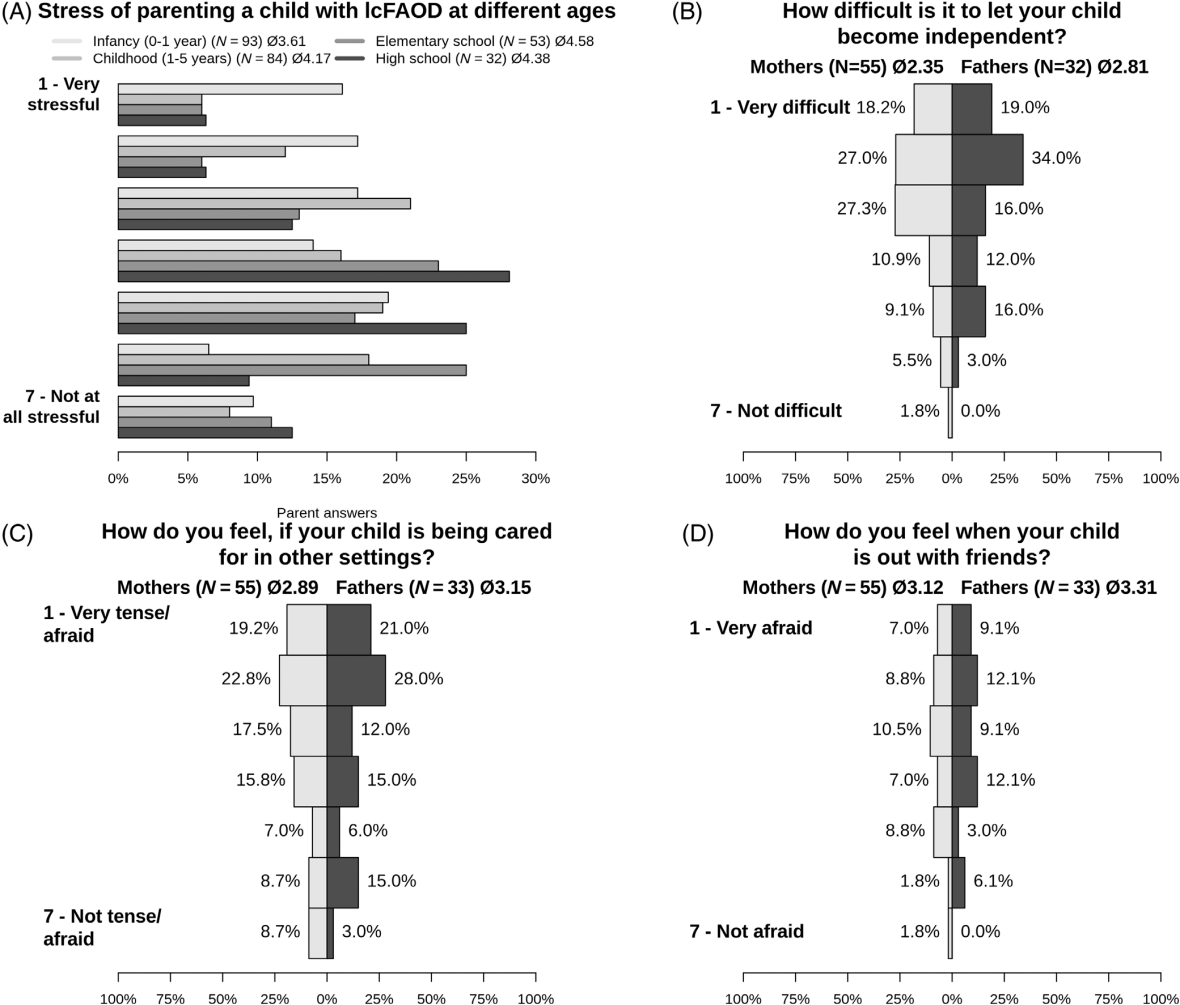
Parental stress related to caring for a child with a lcFAOD and challenges associated with the development of autonomy of the child. Perceived parental stress related to (A) parenting of a child with a lcFAOD at different age groups, (B) development of autonomy (mothers vs. fathers), (C) out‐of‐home care (mothers vs. fathers), and (D) going out with friends (mothers vs. fathers). lcFAOD, long‐chain fatty acid oxidation disorder.

Caregivers were asked to assess how stressful they perceived their life with a child with a lcFAOD at different periods (infancy, early childhood, primary school, and high school age) on a scale from 1 (very stressful) to 7 (not at all stressful) (Figure 3A). The overall parental stress level tended to decrease with increasing age of the child/children, but the perceived stress level of the caregivers was highly individual. When only cases were analyzed in which parental responses were available from both mother and father, no significant differences in the perceived disease burden were detected between mothers and fathers.

### 3.7 Autonomy

The majority of caregivers did not find it difficult to let their child become independent and autonomous (Figure 3B). However, some caregivers reported to feel tense when the child is under external care (Figure 3C) or alone with friends (Figure 3D). Parental concerns included dietetic adherence and compliance with maximal fasting times as well as fear of a sudden metabolic decompensation.

Thirty‐six percent of patients have never stayed overnight at relatives/grandparents, mostly due to parental concerns. Only 39% of patients (*n* = 24) had stayed overnight at a friend's house. However, 37% (*n* = 21) had participated in school trips or youth camps or had been on holiday alone.

### 3.8 Physical activity/sports

**FIGURE 4:**
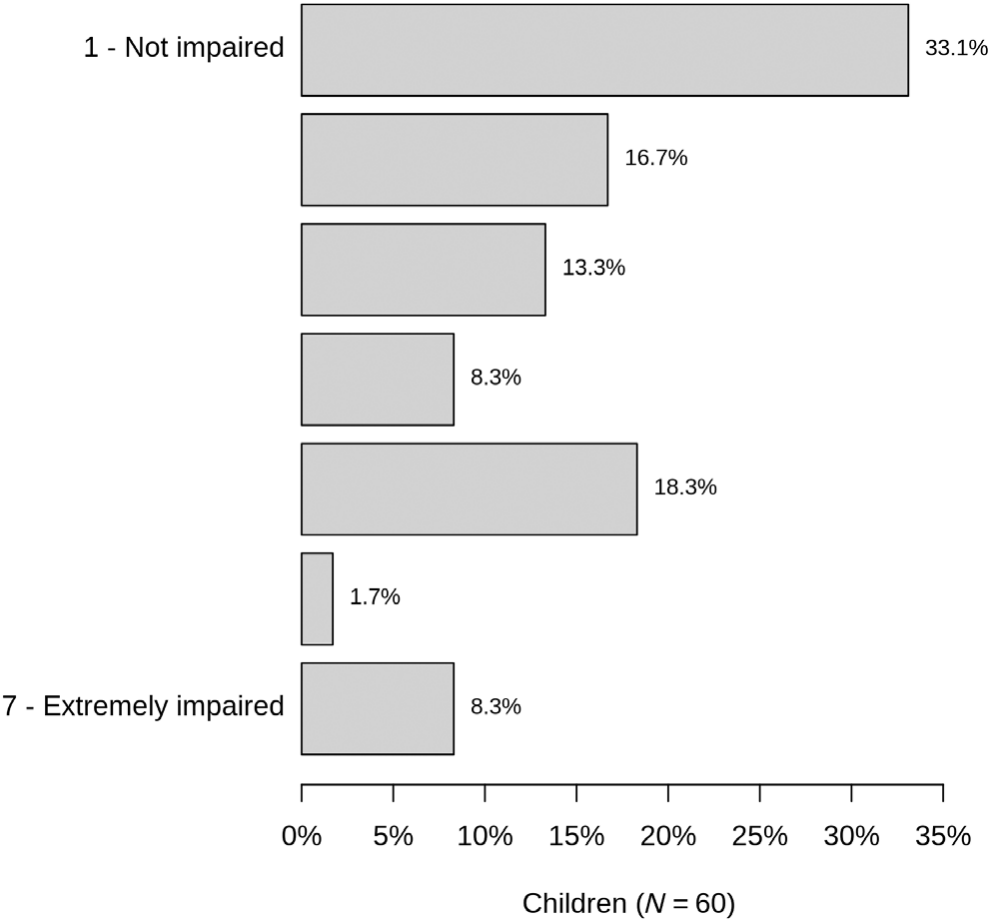
Limitations on their child's physical performance due to the metabolic disorder as perceived by the parents.

Caregivers were asked if the metabolic disease impacts their children's physical performance. In 50% of patients (*n* = 30), parents/caregivers do not consider their child as physically impaired by the lcFAOD (ranks 6–7 on a scale from 1 to 7), while the remainder perceives at least some degree of physical impairment (ranks 1–5 on a scale from 1 to 7) (Figure 4). Only 40% of patients were reported to regularly do sports. About half of these patients do usually not have muscular pain after physical exercise, while the other half reported muscular symptoms after doing sports. Most patients were at least able to regularly take part in school sports. To stay anabolic, the majority either took a carbohydrate‐rich meal or medium chain triglycerides (MCT) oil before physical exercise.

### 3.9 Traveling

**FIGURE 5:**
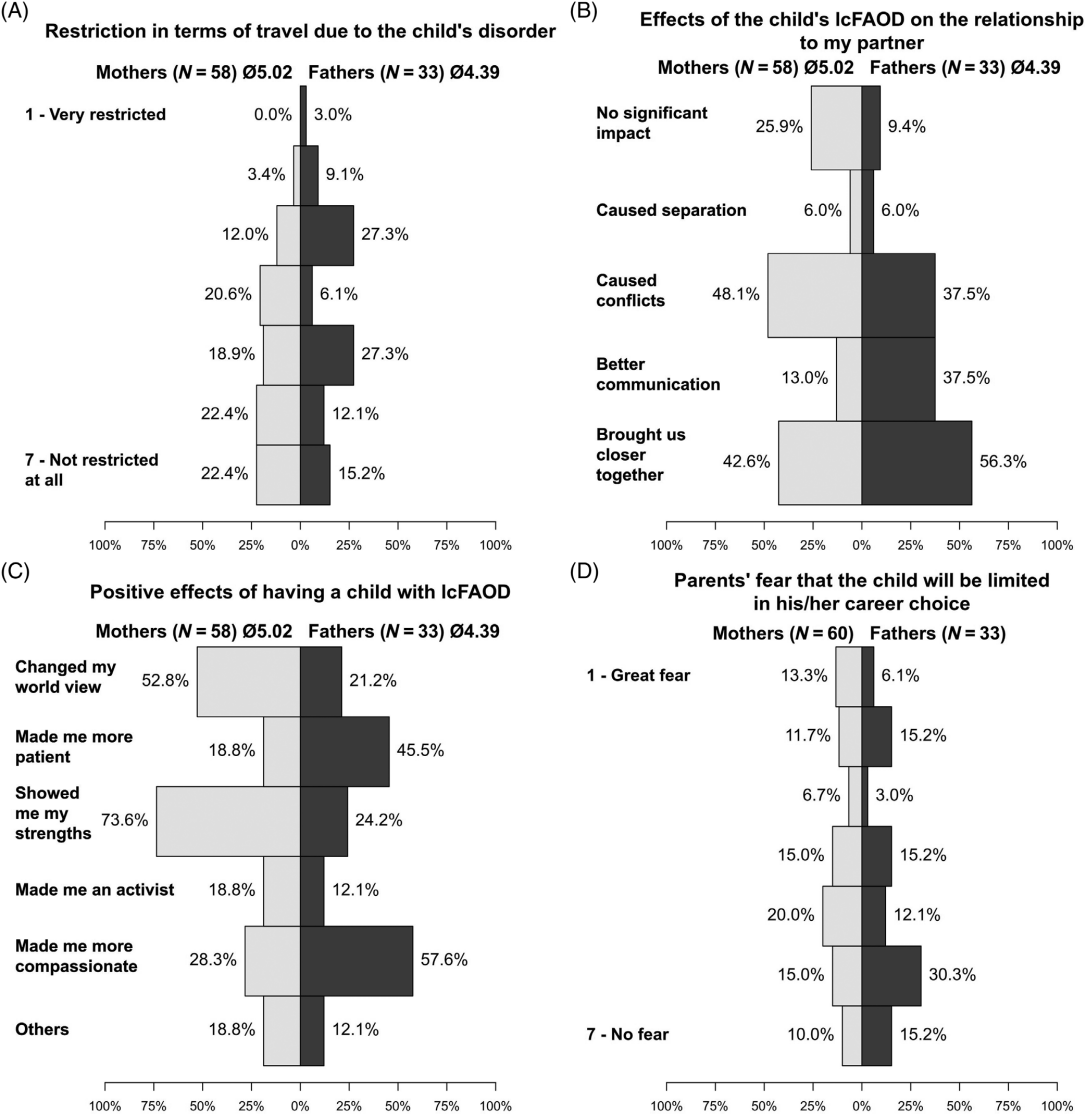
Positive and negative effects of having a child with lcFAODs on different aspects of life perceived by parents/caregivers. (A) Restrictions in terms of travel due to the child's disorder. (B) The impact of having a child with lcFAOD on parental partnership (multiple answers possible). (C) Positive effects of having a child with a lcFAOD (multiple answers possible). The answer “others” included the following answers by fathers: “has brought our family closer together, not taking everything for granted (anymore), enjoyment of small things, more humility in general, better understanding of other parents whose children have special needs, made me proud: our daughter is brave and strong despite her illness”; answers by mothers: “solidarity with other sick people, gratitude for the status quo, my resilience has increased, accepting challenges and responding to them, appreciate family life more, lets me enjoy the day and not get bogged down with unnecessary small stuff, met many new great acquaintances and people, I learned to take my time off to clear my head and started doing sports, made me more relaxed and trained me to accept that there is a lot you don't know (yet), I don't have to understand everything in detail.” (D) Parents'/ caregivers' fear that the child will be limited in his/her career choice due to the metabolic disorder (mothers vs. fathers). lcFAODs, long‐chain fatty acid oxidation disorders.

Many parents/caregivers experience a restriction in terms of travel due to their child's disorder. Again, mothers feel more restricted in this respect than fathers (Figure 5A). Thirteen percent of caregivers reported to only reluctantly travel with their child due to the necessary complex planning (ranks 1–2 on a scale from 1 to 7). Thirty‐two percent experienced traveling as associated with particular stress (rank s1–2 on a scale from 1 to 7). More than 50% considered a hotel vacation with meals as difficult because of their child's special diet (ranks 1–2 on a scale from 1 to 7). Eight families (13%) reported that they had not traveled at all since the diagnosis of their child, and 44% of families had only traveled within Germany. While only a small proportion of parents/caregivers pronounce to be afraid of traveling with their child and feeling insecure when traveling in general (14%, ranks 1–2 on a scale from 1 to 7), more than 85% (ranks 1–2 on a scale from 1 to 7), reported to be afraid of traveling to developmental countries.

### 3.10 Social support

More than three quarters of caregivers reported to have a well‐functioning social network that supports them in daily life (78% of mothers, 77% of fathers; ranks 1–3 on a scale from 1 to 7). Especially, relatives and friends provided support for caregivers, but also nursing services and family helpers provided by official institutions. Nevertheless, 22% of mothers felt left alone with the metabolic disorder of their child, while only 6% of fathers reported that feeling (ranks 5–7 on a scale from 1 to 7). Most psychological support not only came from partners (74%), family (64%) and friends (52%), but also the patient organization and the contact with other affected parents/caregivers played an important role (44%). The latter was considered especially important for providing information on government support options. Many parents/caregivers felt not well supported by state institutions such as the health insurance, care insurance, retirement funds, and pension office.

Fifty‐six percent of patients had a recognized degree of disability (68% of LCHAD deficiency patients, 67% of MTP deficiency patients, 50% of CPT2 deficiency patients, 40% of VLCAD deficiency patients) and 36% had a care degree (56% of MTP deficiency patients, 44% of LCHAD deficiency patients, 24% of VLCAD deficiency patients).

### 3.11 Impact of the child's disease on the parents' professional career/working life

All fathers (100%) and 74% of mothers were employed at the time of the survey, thereof 97% of fathers and 67% of mothers were working full time, respectively. Twelve percent of mothers were on maternity leave. While 36% of mothers reported temporary career breaks, this only applied to 3% of fathers. Eighty‐one percent of fathers felt not professionally restricted by their child's disease, whereas only 48% experienced no professional restrictions (ranks 1–3 on a scale from 1 to 7). Eighty‐five percent of fathers and 48% of mothers reported that their child's disease had no impact on their career choices. In contrast, 9% of fathers and 19% of mothers took a less demanding job than they had originally aimed at. Seven percent of mothers and 0% of fathers quit their job to care for their child with lcFAOD. Fifty‐one percent of mothers and 42% of fathers reported to feel exhausted.

### 3.12 Impact of the disease on family life, parental partnership, and siblings

Eighty‐three percent of parents/caregivers reported to be married, 7% were married, but lived separately, 7% of couples were divorced, 1% were single parents, and 2% were widowed or separated, but lived in a new partnership. In 25% (16) of cases, the patient was the only child of the family, while 33 (52%), 11 (18%), and 3 (5%) patients had 2, 3, and 5 children in their family, respectively. In 52 (82%) families, the patient was the only affected child in the family, in 7 (11%) families 2 siblings were affected, and one family (5%) has 3 affected children.

The impact of the disease of the child on his/her parents'/caregivers' partnership is displayed in Figure 5B. Although the lcFAOD of the child often caused conflicts, many parents/caregivers report, that it also brought them closer together as a couple and led to better communication between the partners. Only 27% of mothers and 29% of fathers were of the opinion that the metabolic disorder of their child represented a burden for the nonaffected sibling(s) (ranks 5–7 on a scale from 1 to 7).

### 3.13 Financial burden

The financial burden for the family due to the lcFAOD of the child is usually not considered high (67% of mothers, 69% of fathers; ranks 1–3 on a scale from 1 to 7). Forty‐seven percent of caregivers report that the additional costs were less than 50€ per month, 33% between 50 and 100€ per month, 18% between 100 and 200€ per month, and in only 2% costs were higher than 200€ per month. In no family, additional costs exceeded the amount of 500€ per month.

### 3.14 Disease burden and disease perception

About 50% of parents/caregivers (48% of fathers and 53% of mothers) reported to think about the implications that the lcFAOD of their child will have on his/her future life at least once per week. These worries include the possible death of their child in 10% of parents/caregivers.

**FIGURE 6:**
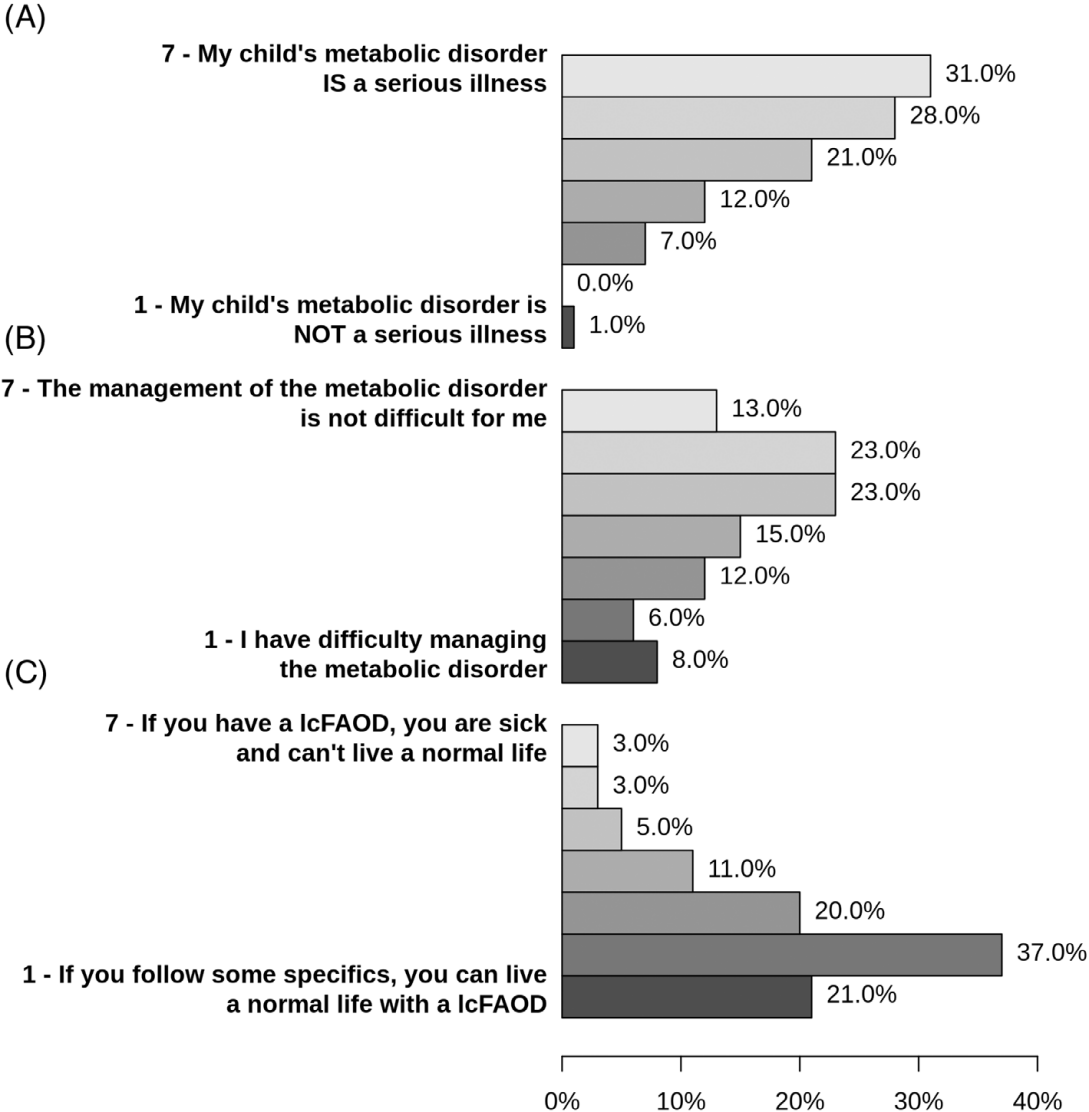
Evaluation of the severity of lcFAOD and the disease burden (A), challenges of the management of the lcFAOD (B), and the possibility to live a “normal life” with a lcFAOD (C). Most parents/caregivers consider lcFAOD a disease a serious disease. Attitude toward the challenges of management was highly variable among parents/caregivers. The majority of patients thought that life with a lcFAOD is well‐manageable and patients with lcFAOD are able to live a normal life if certain measures are taken. lcFAODs, long‐chain fatty acid oxidation disorders.

Figure 6 shows the opinion of parents and caregivers on the severity of their child's disease and the challenges of therapy. More than three quarters of parents/caregivers considered their child's disorder as a serious disease, but the majority also thought that life with a lcFAOD is well manageable. Parents/caregivers were also asked for their opinion on to what extent the metabolic disorder will have an impact on different areas of life of their child in the future. The results are shown in Table 1. Parents/caregivers were particularly concerned about the impact of the lcFAOD on physical fitness, economic consequences, and absences from work/school. Only few parents/caregivers worried about impacts on the intellectual ability of their child. The main burdens expressed by parents/caregivers were emotional stress (67% of fathers and 74% of mothers) and the lack of free time for themselves (49% of fathers and 59% of mothers). Nineteen percent of mothers and 6% of fathers reported a loss of friendships due to their child's disease, and 33% of mothers and 9% of fathers felt isolated. Thirty‐six percent of caregivers reported to have less social contacts, 73% had less time for themselves, 43% less time for siblings, while 19% also felt that they had more time for their children. Parents/caregivers also experienced positive impacts of their child's disease, which are displayed in Figure 5C. Interestingly, mothers mainly reported that the situation has shown them their strengths and has changed their view, while fathers reported that their child's disorder had made them more compassionate and patient.

**TABLE 1:** Estimated impact of the child's FAOD on different areas of life, at present and in the future (“How high do you estimate the impact of your child's illness on different areas of life?”).

	1 (no impact)	2	3	4	5	6	7 (great impact)
Physical fitness	1%	21%	19%	10%	20%	18%	12%
Leisure activities	3%	21%	18%	20%	24%	9%	6%
Friendships	41%	24%	13%	10%	7%	11%	3%
Partnership	28%	21%	16%	11%	12%	5%	7%
Economic impact	4%	31%	13%	16%	18%	9%	9%
Absences at the place of training or work	6%	25%	13%	19%	17%	10%	10%
Intellectual performance	48%	24%	12%	7%	4%	1%	3%
Emotional setting	10%	21%	20%	21%	17%	6%	7%

Abbreviation: FAOD, fatty acid oxidation disorder.

All parents/caregivers communicated their child's disease openly with family members and friends. Only 16% of caregivers received professional psychological support (at least for a certain period).

### 3.15 Parental concerns about the professional future of their child

Caregivers were asked about their concerns with respect to the professional future of their child/children (Figure 5D). On average, mothers were more pessimistic about their child's career choices than fathers. Ranking their concerns that their child would be limited in his/her career choice (1 corresponds to great concerns, 7 corresponds to no concerns), mothers yielded an average rank of 3.76 compared to 4.54 in fathers. Forty‐two percent of mothers and 27% of fathers expressed their concern that their child will not be able to pursue his/her dream job. Limitations were seen primarily with regard to physical fitness, muscular weakness, dietary restrictions, and limited fasting times. In LCHAD and MTP deficiency, retinopathy with loss of vision and neuropathy were also regarded as barriers to the dream job.

## 4 DISCUSSION

Having a child with a chronic disease has a substantial impact on the daily life of parents and caregivers and may seriously affect their own QoL. This is especially true for diseases that are associated with the risk of acute deterioration or decompensations, such as lcFAODs. The aim of this study was to assess the impact of the child's disease on different aspects of daily life of parents and caregivers including work, social and family life. In addition, parents' perception of disease severity and their development of coping strategies were evaluated.

Giving bad news is one of the most challenging forms of clinical communication, and several studies have assessed parent's experiences with the communication of NBS results.^13^, ^14^, ^15^ Abnormal results of NBS for metabolic diseases are known to create substantial distress for parents.^14^ Many parents experience substantial frustration, particularly related to how results are initially communicated and how timely reliable information and clear confirmation of the disease are received.^16^ An Italian study that recently qualitatively evaluated parents' experience of the communication process of positivity at NBS for metabolic diseases demonstrated that parents were largely dissatisfied with the quality and depth of the information received.^17^ According to previous studies, the majority of parents in our study perceived the first communication of the diagnosis as traumatizing. Of course, the timing of the diagnostic opening interview always falls during a very vulnerable period while the young family is still settling into the new family situation. In comparison with many other target diseases of NBS, the work‐up for the confirmation of a lcFAOD usually requires enzyme or genetic testing and therefore, often takes longer than metabolite screening, and waiting for results may exacerbate parents' distress.^14^ In Germany, due to legal and organizational reasons, the diagnosis is often communicated by a physician inexperienced in the field of inborn errors of metabolism who can therefore provide only little information to the parents. Improvements in communications, development of information material for patients, and tailored counseling to meet the needs of culturally and educationally diverse families is needed to reduce parents' distress.^14^, ^15^ Psychological support should be offered early and continuously to guide parents through the diagnostic process and help them to develop a positive perception of their child's disease. It is of note that only 16% of caregivers in our study reported to have received professional psychological support at any time since the birth of their child. Overall, the complex multiprofessional medical and psychosocial care has to be steered by a specialized metabolic center for inborn errors of metabolism.

Caring for a child with a lcFAOD impacts all areas of life, including social and work life. Caregivers are faced with psychosocial, organizational, and financial burdens.^18^ Gender differences were most distinct with respect to work life. While less than 20% of fathers felt professionally restricted by their child's disease, more than 50% of mothers experienced professional restrictions. The same is true for the impact of the child's metabolic disorder on career choices with mothers more often taking a less demanding job than originally aimed at than fathers. Few mothers even quit their job to care for their child with lcFAOD. Nevertheless, employment rates for mothers and fathers taking part in this survey are higher than the average for the German population collected at the Microcensus 2021.^19^ Kish et al. have summarized existing literature on working parents of children with a chronic condition, by focusing on patterns of parental work, the challenges experienced, and the consequences to well‐being.^20^ The authors showed that employment in such parents is less common, and that working parents of chronically ill children experience numerous challenges including balancing work and family, time constraints, stress, and feelings of “doing it all.” These challenges lead to additional impacts on parental QoL.^20^

A child's chronic disease may disrupt family roles, relationships, function and parental involvement in family caregiving.^21^ In addition, healthy siblings of the sick child often become sibling carers and are at risk to become a “shadow child”, who can feel left behind and unseen as families and professionals focus upon their ill sibling.^22^ The experienced impact of the child's lcFAOD on family life of the participants of this study is diverse. While the chronic disease was often the cause of conflicts, many caregivers also reported positive experiences with respect to their parental partnership such as better communication. A negative impact on siblings was only reported by 28% of parents.

A financial burden is reported by a significant proportion of families whose children are diagnosed with a chronic illness.^23^ While the financial burden for diseases with dietary treatment such as celiac disease, phenylketonuria or urea cycle disorders (UCDs) is estimated to be high,^24^, ^25^ participants in our survey see their financial burden as rather low. Compared to the financial burden that US families with UCD already reported 20 years ago with mostly over 100 to over 1000 US$ per month,^23^ the financial expenses of lcFAOD families in Germany and Austria do usually not exceed 200€ per month. This is probably because most MCT‐containing products such as formula, oil, and margarine are reimbursed by health insurances in Germany. Also, additional therapies such as speech therapy, physiotherapy, and occupational therapy are usually covered by health insurance, at least during childhood and adolescence.

Parents and caregivers take primary responsibility for their child's disease. Dietary management and monitoring as well as handling emergency situations can be challenging in lcFAOD, and parents live with a constant fear of metabolic crisis and neurological sequelae. A recent study by Bösch et al. could show that both caregiver burden and disease severity perception of parents play a crucial role for the health‐related QoL of pediatric patients with intoxication‐type inborn errors of metabolism.^4^ In our study, more than three quarters of caregivers considered their child's disorder as a serious disease. Nevertheless, the majority also thought that life with a lcFAOD is well manageable. Disease perception is highly subjective and does not always reflect the severity of the disease. Gramer et al. have assessed parents' perspectives on child development and social impact on families of 187 patients with metabolic disorders detected by NBS.^26^ In this study, parents rated the development of their child, the perceived burden on child and family, and future expectations by standardized questionnaires. Interestingly, disorders rated as potentially very burdensome by experts were not rated accordingly by parents, demonstrating different perspectives of professionals and parents and showing that disease burden is not equivalent with objective features such as complexity of treatment, risk for metabolic decompensation, and outcome.^26^

In our study, the overall parental stress level tended to decrease with increasing age of the child. This may reflect that families adapt well to their child's diagnosis and probably develop successful coping strategies. A Canadian study that used semi‐structured telephone interviews with parents and caregivers of patients with inherited metabolic diseases revealed that parents develop proactive coping strategies to integrate complex disease management protocols into routine family life.^27^ Nevertheless, many parents in our study worried about the future of their child, especially with respect to career choices. Parental concerns focused on metabolic crises, muscular symptoms, dietary restrictions, and irreversible retinopathy and neuropathy in patients with LCHAD/MTP deficiency. In the study by Bösch et al., especially parents' severity assessment of their children's disease appeared to be an important predictor of patients' health‐related QoL.^4^ This underlines the need for comprehensive multidisciplinary care of lcFAOD patients and families including psychological and social support.^26^ As suggested by Bösch et al., interventions targeting disease perception could be promising options to support parents who experience high caregiver burden to reduce negative consequences for their children.^4^ Patient support groups may also provide substantial support and empower patients and families in their daily life to cope with the child's disease. Patients in this study mainly experienced the patient organization as an important source of emotional and practical support. Interestingly, in contrast to common diseases such as diabetes, there is no trial evidence on the benefits of patient support groups for rare diseases.^28^ In a scoping review including 10 publications on rare disease patient support groups, Delisle et al. identified seven different perceived benefits for participants: (1) meeting and befriending other people with the same rare disease and similar experiences; (2) learning about the disease and related treatments; (3) giving and receiving emotional support; (4) having a place to speak openly about the disease and one's feelings; (5) learning coping skills; (6) feeling empowered and hopeful; and (7) advocating to improve healthcare for other rare disease patients.^28^ To maximize the benefits for patients participating in rare disease patient groups, doctors and patients should work together to enhance access to support groups and to share information on all important medical issues and psychosocial aspects.

In conclusion, having a child with a lcFAOD poses a significant burden on the daily life of parents and caregivers; however, most parents have a positive attitude toward their child's disease and seem to cope well with their situation. The identification not only of the patients but of the parents' needs helps in further improving multidisciplinary care and the further institution of metabolic centers for patients with inborn errors of metabolism which provide immediate and expert consultation after a positive NBS or a clinical diagnosis for parents and caregivers.

## AUTHOR CONTRIBUTIONS

*Conceptualization*: Maren Thiel and Sarah C. Grünert. *Questionnaire development*: Maren Thiel, Sarah C. Grünert, and Stefanie Rosenbaum‐Fabian. *Dissemination of the questionnaire and recruitment*: Maren Thiel and Sarah C. Grünert. *Data analysis*: Maren Thiel, Sven F. Garbade, and Sarah C. Grünert. *Manuscript draft*: Sarah C. Grünert and Maren Thiel. *Draft of figures*: Maren Thiel, Sarah C. Grünert, and Sven F. Garbade. *Revision of the manuscript*: Maren Thiel, Sven F. Garbade, Stefanie Rosenbaum‐Fabian, Ute Spiekerkoetter, and Sarah C. Grünert.

## FUNDING INFORMATION

No funding was received for this work.

## CONFLICT OF INTEREST STATEMENT

M.T. acts as chairwoman of the German patient organization for patients with fatty acid oxidation disorders (Fett‐SOS e.V., https://www.fett-sos.com/). She also participates as a patient representative in a patient leadership council by Ultragenyx GmbH. SFG and SR‐F declare no conflicts of interest. U.S. and S.C.G. declare participation in Ultragenyx Clinical Studies in glycogen storage diseases and fatty acid oxidation defects (with all funding paid to the institution) as well as adboard participation for Ultragenyx GmbH. U.S. was a member of the Nutricia Milupa EMG Advisory Board (temporary member 2022–2026, unpaid). S.C.G. declares lecture honoraria from Vitaflo GmbH and honoraria from Danone GmbH for the development of patient information material outside the submitted work.

## ETHICS STATEMENT

This study has been approved by the ethics committee of the University Hospital Freiburg (EKFR Nr. 20–1132).

## CONSENT FOR PUBLICATION

All participants gave their consent for participation in this study.

## Supporting information


**Data S1.** Supporting information.

## Data Availability

The datasets used and/or analyzed during this study are available from the corresponding author on reasonable request.
