# Supplementary material for: Psychosocial issues and coping strategies in families affected by long‐chain fatty acid oxidation disorders
Source: JIMD Rep. 2023 Dec 5;65(1):25–38. doi: 10.1002/jmd2.12402 (PMC10764200; doi:10.1002/jmd2.12402)
Supplement: Supplementary file 1 — Data S1. Supporting information. [file JMD2-65-25-s001.pdf]

ID-Nr.___________________

**You can use the pictograms to see where you are in the questionnaire**


| | Topic | Page |
|---|---|---|
| | *A - Diagnosis* | *Page 4* |
| | *B - Infancy* | *Page 7* |
| | *C - Toddlerhood* | *Page 9* |
| | *D - School age* | *Page 13* |
| | *E – occupational training* | *Page 18* |
| | *F - Diet and everyday life* | *Page 20* |
| | *G - Clinical care* | *Page 25* |

| | | |
|---|---|---|
| | *H - Independence* | *Page 28* |
| | *I - Sport/ physical activity* | *Page 30* |
| | *Travel* | *Page 23* |
| | *Social support* | *Page 24* |
| | *Parental occupation* | *Page 25* |
| | *Family life* | *Page 27* |
| | *General information* | *Page 29* |
| | *Effects on life* | *Page 30* |


**Z - Social demographic data**

**Z.1 I am myself**

☐ biological mother of a patient

☐ biological father of a patient

☐ others (e.g. stepmother/father, foster parents,...): _______________

**Z.2. age of the patient _____________** years

**Z.3. sex of the patient**

☐ female ☐ male

**Z.4.1 What type of long-chain fatty acid oxidation disorder does your child have?**

☐ VLCAD deficiency ☐ MTP deficiency ☐ CACT deficiency

☐ LCHAD deficiency ☐ CPT1 deficiency ☐ CPT2 deficiency

**Z.4.2 If VLCAD deficiency: Is the residual enzyme activity known?**

☐ yes ☐ no

**Z.4.3 If yes, what percentage?** _________________%

**Z.4.4 If LCHAD/MTP deficiency: My child has a proven neuropathy**

☐ yes ☐ no ☐ not applicable

**Z.4.5 If LCHAD/MTP deficiency: My child has a proven retinopathy**

☐ yes ☐ no ☐ not applicable

**Z.5.1 How many children do you have?**

Number of children: _________________

**Z.5.2 How many children in the family have a long-chain fatty acid oxidation disorder?**

Number of children with a FAOD: _________________

**Z.6 What is your nationality?**

☐ German ☐ Polish

☐ Austrian ☐ Russian

☐ Turkish ☐ other __________________

☐ Arabic

**Z.7. marital status of the parents**

☐ married/partnership and live with my spouse or partner

☐ married/partnership and live separately from my spouse/partner

☐ Divorced

☐ Single and single parent

☐ other __________________________

**Z.8 Pseudonymization**

First letter Place of birth of first-born child, mother and father and own sex A = female, B = male)

(e.g. Dresden, Bautzen, Berlin, mother = DBBA)

Pseudonym: _________________________

## A - DIAGNOSIS*:*

**A.1 When was your child suspected of having a long-chain fatty acid oxidation disorder?**

☐ through the newborn screening
☐ at the age of ____________________________

**A.2 When was the presence of a long-chain fatty acid oxidation disorder confirmed in your child?**

☐ through confirmation diagnostics immediately after newborn screening (e.g. control screening)
☐ at the age of ____________________________

**A.3 At the time of diagnosis, the following existed**
*Multiple answers possible*

☐ No clinical symptoms
☐ Hypoglycemia
☐ Muscle weakness
Gait disturbance
☐ Heart failure
☐ Liver enlargement/ increased liver values
☐ other: ________________________

**A.4.1 Was psychological support offered at the time of diagnosis?**
☐ yes ☐ no

**A.4.2 If yes, have you made use of it**?
☐ yes ☐ no

**A.5.1 From my point of view, the initial diagnostic interview is...**

| | 1 | 2 | 3 | 4 | 5 | 6 | 7 | |
|---|---|---|---|---|---|---|---|---|
| ...went badly | | | | | | | | ...went well |

**A.5.2 Who conducted the initial diagnostic interview with you?**
*Multiple answers possible*

☐ Ward doctor
☐ Metabolic physician
☐ Nutritionist
☐ Psychologist
☐ Human geneticist
☐ others: _________________________

**A.5.3 What did you miss?**

_______________________________________________________________________________

_______________________________________________________________________________

**A.5.4 What was good?**

______________________________________________________________________________

______________________________________________________________________________

______________________________________________________________________________

**A.6 From my point of view, the initial interview (=diagnosis opening interview) was**

*Multiple answers possible*

☐ Encouraging

☐ perspective-opening

☐ discouraging

☐ hopeless

☐ overwhelming

☐ other:____________________

**A.7 What was your first reaction to your child's diagnosis?**

*Multiple answers possible*

☐ Fear

☐ Anger

☐ Feelings of guilt

☐ Disappointment

☐ Apprehension

☐ Relief

☐ Sadness

**A.8 What other sources of information did you use after the initial interview?**

*Multiple answers possible*

| | used | not used | Helpful | not helpful |
|---|---|---|---|---|
| Internet, which page? | | | | |
| Books | | | | |
| Friends/acquaintances | | | | |
| Brochure for parents (e.g. Milupa, Vitaflo, Dr.Schär,...) | | | | |
| Self-help association | | | | |
| Alternative practitioner/alternative medicine practitioner | | | | |
| others:________________________________________ | | | | |

**B - INFANCY (0-1 year)**

**B1. I found the nutrition in infancy to be ....**

| | 1 | 2 | 3 | 4 | 5 | 6 | 7 | |
|---|---|---|---|---|---|---|---|---|
| very problematic | | | | | | | | Not problematic at all |

**B2.1 In infancy, my child could be fully orally fed (bottle-fed/ breastfed).**

☐ yes ☐ no

**B2.2 If no:**

☐ My child sometimes had to be probed (e.g. for a few days, during and after hospital stays).

☐ My child had to be fully probed.

☐ My child was given continuous night-time probes.

**B3. If necessary, probing was carried out using a:**

☐ Nasal probe ☐ PEG tube ☐ not applicable

**B4. Reason/reason for inserting a PEG tube or permanent nasal tube:**

☐ Refusal of food in the context of an acute illness/metabolic imbalance

☐ Insufficient food/calorie intake

☐ Feeding disorder

☐ Permanently elevated blood values (e.g. CK, liver values)

☐ sonstiges:_______________________________________________________________

☐ Not applicable

**B5. The presence of the PEG tube or permanent nasal tube has:**

☐ has eased the feeding situation for me

☐ I found it an additional burden

☐ Not applicable

**B6.1 Were inpatient stays necessary in infancy due to acute illness/need for treatment (e.g. fever, infection, vaccination, metabolic derailment)?**

☐ yes ☐ no

**B6.2 If yes, how many?**

In infancy (up to the 1st day of birth), ________ inpatient stays were required

**B7.1 My child needed special support as an infant:**

☐ yes ☐ no

**B7.2.If yes:**

☐ Physiotherapy

Occupational therapy

☐ Speech therapy

☐ other: _________________________

**B8. I perceived my child's infancy as ...**

| | 1 | 2 | 3 | 4 | 5 | 6 | 7 | |
|---|---|---|---|---|---|---|---|---|
| very stressful | | | | | | | | Not stressful at all |

**B9. I found the following to be the greatest burden in infancy:**

______________________________________________

______________________________________________

______________________________________________

**B10. I experienced the following as positive during infancy:**

______________________________________________

______________________________________________

______________________________________________

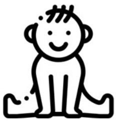

**C – TODDLERHOOD (1-6 years)** *if not yet applicable, continue with "E" (p.18)*

**C1. I found the nutrition in toddlerhood to be ....**

| | 1 | 2 | 3 | 4 | 5 | 6 | 7 | |
|---|---|---|---|---|---|---|---|---|
| very problematic | | | | | | | | Not problematic at all |

**C2.1 In toddlerhood my child could be fully orally nourished**

☐ yes ☐ no

**C2.2 If no,**

☐ My child sometimes had to be probed (e.g. for a short time, for a few days, during and after hospital stays).

☐ My child had to be fully probed.

☐ My child was given continuous night-time probes.

**C3.2 At the toddler age of 1-3 years...**

☐ My child was looked after exclusively by the parents at home.

☐ My child was looked after by a childminder.

☐ My child attended a regular kindergarten:

- ☐ With integration status.
- ☐ Without integration status.

☐ My child attended an inclusive kindergarten:

- ☐ with special support/accompaniment (e.g. individual case workers).
- ☐ with integration status.
- ☐ without integration status.
- ☐ as a regular child without integration status.

**C3.2 If care was provided outside the parental home (kindergarten, day care):**

☐ all day (>7h per day)

☐ half-day (4-7h daily)

☐ by the hour (<4h per day)/ by the day

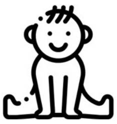

**C4. How was lunch provided in out-of-home care?**

☐ We had to cook lunch for our child at home and bring it to daycare.

☐ At the daycare center/daycare, my child was cooked according to his/her nutrition plan.

☐ My child could not eat lunch at the daycare center/daycare.

**C.5.1 Were there any fears/concerns/reservations regarding the illness on the part of the educator/day-care parent before admission to out-of-home care?**

☐ yes ☐ no

**C5.2 If yes, which ones?**

*Multiple answers possible*

☐ High support costs

☐ Wrong reaction/fear of making a mistake

☐ Nutrition in general

☐ Sonstiges:_____________________________________________________________

**C6.1 Were there any fears on your part as parents before admission to the kindergarden/ daycare center?**

☐ Yes ☐ no

**C6.2 If yes, which ones?**

_______________________________________________________________

_______________________________________________________________

_______________________________________________________________

**C7.1 Were inpatient stays necessary in toddlerhood due to acute illness/need for treatment (e.g. fever, infection, vaccination, metabolic derailment)?**

☐ yes ☐ no

**C7.2 If yes, how many?**

In toddlerhood (from the 1st to the 6th birthday), ________ inpatient stays were necessary

ID-Nr.___________________

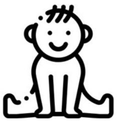

**C8.1 My child needed special support as an toddler:**

☐ yes ☐ no

**C8.2.If yes:**

☐ Physiotherapy ☐ Occupational therapy

☐ Speech therapy ☐ other: ________________________

**C9. I experienced my child's toddler years as ...**

| | 1 | 2 | 3 | 4 | 5 | 6 | 7 | |
|---|---|---|---|---|---|---|---|---|
| very stressful | | | | | | | | Not stressful at all |

**C10.1 Is/was your child teased at kindergarten because of his/her metabolic disorder?**

☐ yes ☐ no

**C10.2 Is/was your child excluded in kindergarten because of his/her metabolic disorder?**

☐ yes ☐ no

**C10.3 If yes (one or both: teased/excluded), how?**

____________________________________________

____________________________________________

____________________________________________

**C11.1 Has your child had positive experiences in kindergarten because of his or her metabolic disorder?**

☐ yes ☐ no

**C11.2 If yes, which one?**

____________________________________________

**C12. I found the following to be the greatest burden in infancy:**

____________________________________________

**C13. I experienced the following as positive when I was a toddler:**

____________________________________________

ID-Nr.__________________

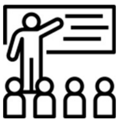

**D - SCHOOL AGE (from 6 years)**

☐ Not yet applicable *<u>If not yet applicable, continue with "E"</u>*

**D1. My child attends the following school(s): (***Multiple answers possible)*

☐ Standard elementary school

- ☐ With integration status
- ☐ With individual case helpers

☐ Integrative school

☐ Special school/ school with a special educational focus:

____________________________________________________________

☐ middle school (desired qualification: Hauptschulabschluss)/ comprehensive school (desired qualification: intermediate school leaving certificate)

☐ Grammar school (desired qualification: general higher education entrance qualification)

☐ others:_________________________________________________

**D2. How was lunch provided at elementary school?**

☐ We had to cook lunch for our child at home and take it to elementary school.

☐ At elementary school, my child was cooked according to his diet plan.

☐ My child did not eat lunch at elementary school.

**D3. How was lunch provided at secondary school?**

☐ We had to cook lunch for our child at home and take it to secondary school.

☐ At secondary school, they cooked for me according to his diet plan.

☐ My child did not eat lunch at secondary school.

☐ Not yet applicable.

**D4.1 Were inpatient stays necessary at school age due to acute need for treatment?**

☐ yes ☐ no

**D4.2 If yes, how many on average per year?**

At school age (6-16 years), an average of ________ inpatient stays were required each year.

**D4.3 My child has (had) absences from school due to routine check-up appointments (specialists, metabolic outpatient clinic)**

☐ none

☐ At least once a month

☐ At least once every four months

☐ At least once every six months

☐ At least once a year

**D4.4 My child has (had) absences from school due to hospitalization for acute illness:**

☐ none

☐ on average _______ days per year

**D.5.1 School attendance - elementary school**

*Multiple answers possible*

☐ My child has openly communicated his or her illness at school with classmates.

☐ My child does not want/would not want his classmates to know about his illness.

☐ My child often feels excluded by their illness.

☐ My child cannot/could not participate in some class activities due to his/her illness.

**D5.2 I myself felt that my child's primary school age/primary school years were ....**

| | **1** | **2** | **3** | **4** | **5** | **6** | **7** | |
|---|---|---|---|---|---|---|---|---|
| very stressful | | | | | | | | Not stressful at all |

**D5.3 Is/was your child teased at elementary school because of his/her illness?**

☐ yes

☐ no

**D5.4 Is/was your child ostracized at elementary school because of his/her illness?**

☐ yes ☐ no

**D.5.5 If yes (one or both: teased/excluded), how?**

______________________________________________

______________________________________________

______________________________________________

**D.6.1 School attendance - secondary school**

*If not yet applicable, please continue with question D7.1.*

*Multiple answers possible*

☐ My child has openly communicated his or her illness at school with classmates.

☐ My child does not want/would not want his classmates to know about his illness.

☐ My child often feels excluded by their illness.

☐ My child cannot/could not participate in some class activities due to his/her illness.

**D6.2 I myself found my child's school age/school years (secondary school) to be ....**

| | 1 | 2 | 3 | 4 | 5 | 6 | 7 | |
|---|---|---|---|---|---|---|---|---|
| very stressful | | | | | | | | Not stressful at all |

**D6.3 Is/was your child teased at secondary school because of his/her metabolic disorder?**

☐ yes ☐ no

**D6.4 Is/was your child ostracized at secondary school because of his/her illness?**

☐ yes ☐ no

**D.6.5 If yes (one or both: teased/excluded), how?**

______________________________________________

______________________________________________

______________________________________________

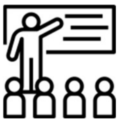

**D7.1 Has your child had positive experiences at school due to his or her metabolic disorder?**

☐ yes ☐ no

**D.7.2 If yes, which ones?**

______________________________________________

______________________________________________

______________________________________________

**D.8.1.I have the impression that my child perceives/has perceived his/her metabolic disorder as ... at school age.**

| | 1 | 2 | 3 | 4 | 5 | 6 | 7 | |
|---|---|---|---|---|---|---|---|---|
| very stressful | | | | | | | | Not stressful at all |

**D8.2 I found the following to be the greatest burden at school age:**

______________________________________________

______________________________________________

______________________________________________

**D8.3 I experienced the following as positive during my school years:**

______________________________________________

______________________________________________

______________________________________________

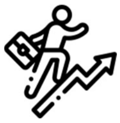

## E - OCCUPATIONAL TRAINING/ FUTURE OCCUPATIONAL TRAINING

**E.1 My child is completing**

☐ an apprenticeship

☐ A degree course

☐ other: ________________________

☐ not yet applicable

**E.2 I have the impression/fear that my child's choice of career due to his/her illness ...**

| | 1 | 2 | 3 | 4 | 5 | 6 | 7 | |
|---|---|---|---|---|---|---|---|---|
| is/will be very limited. | | | | | | | | is/will not be restricted at all |

**E.3.1 I am worried that my child will not be able to pursue his or her dream job due to his or her illness.**

☐ yes

☐ no

This is/would have been: _____________________________________

**E.3.2 If yes, why/due to what restriction**?

______________________________________________________________

______________________________________________________________

______________________________________________________________

**E.4 I think/believe that in adulthood (or from the age of 16) my child will...**

| | 1 | 2 | 3 | 4 | 5 | 6 | 7 | |
|---|---|---|---|---|---|---|---|---|
| is/will be very impaired. | | | | | | | | is/will not be affected at all |

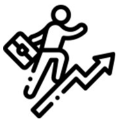

**E.5 I have the impression/anxiety that my child experiences/will experience his/her illness in everyday adult life as.....**

| | 1 | 2 | 3 | 4 | 5 | 6 | 7 | |
|---|---|---|---|---|---|---|---|---|
| very stressful | | | | | | | | Not stressful at all |

**E.6 I feel/assume that the greatest limitation in adulthood is for my child:**

______________________________________________

______________________________________________

______________________________________________

**F - DIET AND EFFECTS ON EVERYDAY LIFE**

**F1. My child is currently following the following diet:**

☐ Strictly low-fat diet (with calculation of the intake of long-chain fats) and MCTs

☐ Strictly low-fat diet (with calculation of the intake of long-chain fats) without MCTs

☐ Moderate low-fat diet (with calculation of the intake of long-chain fats) and MCTs

☐ Moderate low-fat diet (with calculation of the intake of long-chain fats) without MCTs

☐ No diet

**F2.1 My child currently has a feeding tube**

☐ yes ☐ no

**Q.2.2 If yes, which ones:**

☐ PEG tube ☐ Nasal tube

**F3. My child is currently (***multiple answers possible)*

☐ completely orally nourished. ☐ Fully probed.

☐ Partly probed during the day. ☐ only probed at night.

**F4. At night, my child currently gets** *(multiple answers possible)*

☐ a continuous sounding with:

- ☐ Basic -f
- ☐ Basic -f + MCT oil
- ☐ Monogen/ Lipistart/ Lipano as a permanent probe
- ☐ Maltodextrin as a permanent probe
- ☐ other:_______________________________________________

☐ Quantity:___________ Night meal(s) with:

- ☐ Basic f
- ☐ Basic -f + MCT oil
- ☐ Mondamin/ Glycosade
- ☐ Monogen/ Lipistart/ Lipano
- ☐ other: _______________________________________________

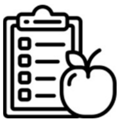

**F5. My night is currently interrupted due to necessary meals__________ times**

**F6. How do you currently organize the night meals?**

☐ My child eats his/her night meals independently.

☐ I am solely responsible for the night meals.

☐ We alternate feeding at different times.

☐ We alternate feeding on a daily basis.

☐ We alternate feeding on a weekly basis.

☐ others: ___________________________________________________________

**F7.1 The interrupted night's sleep leads to...**

| | 1 | 2 | 3 | 4 | 5 | 6 | 7 | |
|---|---|---|---|---|---|---|---|---|
| severe sleep disorders. | | | | | | | | no sleep disorders at all. |

**Q7.2.Due to the interrupted night's sleep I feel**

| | 1 | 2 | 3 | 4 | 5 | 6 | 7 | |
|---|---|---|---|---|---|---|---|---|
| still fully efficient. | | | | | | | | not fully efficient. |

**F8.1 Our family is responsible for preparing low-fat meals**

*(multiple answers possible)*

☐ Mother

☐ Father

☐ my child itself

☐ others:__________________________

**Q8.2 At what age did you start including your child in the diet?**

☐ with___________years ☐ not yet applicable

**Q9.1.I feel that monitoring/controlling compliance with my child's special diet is ...**

| | 1 | 2 | 3 | 4 | 5 | 6 | 7 | |
|---|---|---|---|---|---|---|---|---|
| Not stressful at all | | | | | | | | very stressful |

**Q9.2 When feeding my child and adhering to the diet...? (e.g. not always cooking only the desired food, fat reduction)**

| | 1 | 2 | 3 | 4 | 5 | 6 | 7 | |
|---|---|---|---|---|---|---|---|---|
| I am sufficiently consistent. | | | | | | | | I am not consistent. |
| I am satisfied with my consistency | | | | | | | | I would like to be more consistent. |

**Q10.1 Does your child eat outside ... ?** (*multiple answers possible*)

☐ in the daycare center/school

☐ with relatives

☐ with friends

☐ No, my child never eats outside

**Q10.2 Do you normally have to provide meals when eating out?**

☐ yes

☐ no

**Q10.3.Do you eat out with your child in a restaurant (including fast food outlets)?**

☐ at least once a week

☐ one to three times a month

☐ Less than once a month

☐ No, never before

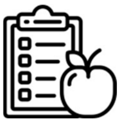

**Q10.4 Is eating in a restaurant usually...?**

| | 1 | 2 | 3 | 4 | 5 | 6 | 7 | |
|---|---|---|---|---|---|---|---|---|
| unproblematic, there is always a dietary suitable/adaptable meal. | | | | | | | | very problematic, there is never a dietary suitable/adaptable food. |

**F11.1 We try to treat minor infections (colds, high temperature) at home.**

| | 1 | 2 | 3 | 4 | 5 | 6 | 7 | |
|---|---|---|---|---|---|---|---|---|
| rare | | | | | | | | often |

**Q11.2 Do you use a diet plan/emergency plan at home for such cases in the event of minor illnesses with fever?**

☐ yes ☐ no

**F11.3 We present ourselves at the clinic at an early stage, even for minor infections**

☐ yes ☐ no

**F11.4 We go to the clinic for the following symptoms:**

*(multiple answers possible)*

☐ Vomiting

☐ Diarrhea

☐ Fever

☐ Restricted appetite/refusal to eat

☐ Feeling unwell

☐ Muscle pain

☐ other: ________________________

**Q12 Does your child have a port/ or a permanent central venous catheter, e.g. CVC, Hickman, etc.?**

☐ yes, current ☐ no

☐ Currently no, but in the past

ID-Nr.____________________

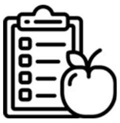

**Q13.1 Is your child vaccinated?**

☐ Yes, all vaccinations on time according to STIKO guidelines (official recommendations of the Standing Committee on Vaccination).

☐ Yes, but with an individual vaccination plan.

☐ No.

**Q13.2 Have you taken special precautions for the vaccinations?**

*(multiple answers possible)*

☐ Inpatient admission for the (first) vaccinations.

☐ Precautionary antipyretic measures (ibuprofen, paracetamol).

☐ Precautionary nutrition according to the emergency plan for 1-2 days around the vaccination.

☐ No.

**Q13.3 Has your child ever had any problems in connection with vaccinations?**

☐ yes ☐ no

**Q13.4 If yes, which one**?

______________________________________________

______________________________________________

______________________________________________

## G - CLINICAL CARE

**G1. How often do you regularly present your child to the metabolic outpatient clinic at this stage of life?**

☐ regularly more than every 3 months

☐ regularly every 3 months

☐ regularly every 6 months

☐ regularly once a year

☐ regularly less than once a year

☐ irregular

☐ Only if requested by the metabolic clinic

☐ Not at all

**G2.1 Do you feel well informed about your current examination results (e.g. laboratory results, ultrasound)?**

☐ yes

☐ no

**G2.2 If "NO", why not?**

☐ I am not informed of the results.

☐ The results are communicated to me, but not explained.

☐ The results are communicated to me but I don't understand them.

☐ I don't care about the results.

**G3.1 Is your child afraid of going to the outpatient clinic?**

☐ yes, current

☐ No, never before

☐ yes earlier, up to the age of _____________ years

**G3.2 If "YES", what was/were he/she afraid of when presenting at the outpatient clinic?**

*(multiple answers possible)*

☐ in front of the doctors in general

☐ before the blood sample is taken

☐ before examinations such as EEG/EMG/NLG

☐ before ultrasound examinations (abdominal sono, cardiac echo)

☐ before other examinations: ______________________________

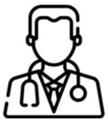

**G.4.1 Who controls the treatment of your child during inpatient stays?**

*(multiple answers possible)*

☐ Your attending metabolic physician or ward physician with consultation of the metabolic outpatient clinic

☐ Ward physicians without consulting the metabolic physician in charge

☐ We are involved in therapy planning (e.g. discharge date)

☐ Support is provided by the nutritionist/dietician

**G4.2 Do you use the free choice of doctor or have you ever obtained a second medical opinion on your child's treatment?**

☐ yes ☐ no

**G5.1 How often do you receive nutritional advice?**

☐ At every presentation in the metabolic outpatient clinic.

☐ If we wish to do so.

☐ Sporadic.

☐ Sonstiges:____________________

**G5.2 I have a nutritionist responsible for us who I can contact at any time if I have questions about my child's diet?**

☐ yes ☐ no

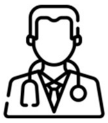

**G5.3 How do you feel about the nutritional advice?**

| | 1 | 2 | 3 | 4 | 5 | 6 | 7 | |
|---|---|---|---|---|---|---|---|---|
| Helpful for the daily implementation of the diet. | | | | | | | | Not helpful for the daily implementation of the diet. |
| Helpful for determining the current nutritional requirements (amount of energy, calories) | | | | | | | | Not helpful for determining current nutritional requirements (amount of energy, calories) |
| Helpful for determining the amount of LC fats, MCT fats and essential fatty acids. | | | | | | | | Not helpful for determining the amount of LC fats, MCT fats and essential fatty acids. |
| I always receive information about new diet products. | | | | | | | | I never get information about new diet products. |
| Very helpful overall. | | | | | | | | Overall not helpful. |

**G5.5 Is there anything else you would like to tell us about nutritional advice?**

___________________________________________________________________

___________________________________________________________________

___________________________________________________________________

ID-Nr.___________________

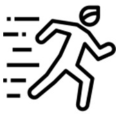

## H - INDEPENDENCE

**H1. How do you feel about letting your child become independent?**

| | 1 | 2 | 3 | 4 | 5 | 6 | 7 | |
|---|---|---|---|---|---|---|---|---|
| I don't find it difficult to let my child become independent. | | | | | | | | I find it very difficult to let my child become independent. |

☐ Not yet applicable (small child)

**H2.1 At what age did your child first stay overnight with other family members, e.g. grandparents (without you)?**

☐ _________ years

☐ No, my child has never spent the night with grandparents or similar

**H2.2 If "NO" why not?**

☐ Concerns on the part of parents

☐ Concerns on the part of family members/grandparents etc.

☐ Has not yet arisen/was not yet necessary

☐ not possible

**H3.1 At what age did your child stay overnight with friends (without you) for the first time?**

☐ _________________Years

☐ No, my child has never spent the night alone with friends

**H3.2 If "NO", why not?**

☐ Concerns on the part of parents

☐ Concerns on the part of friends/parents of friends

☐ has not yet occurred

ID-Nr.____________________

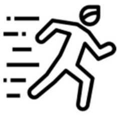

**H4. How do you feel when your child is temporarily looked after somebody else?**

| | 1 | 2 | 3 | 4 | 5 | 6 | 7 | |
|---|---|---|---|---|---|---|---|---|
| I am not tense when my child is being looked after somebody else. | | | | | | | | I am very tense when my child is being looked after somebody else. |

**H5.1 Has your child ever been on a school trip or youth camp alone (without parents) or gone on vacation alone?**

☐ yes ☐ no

**H.5.2 If yes, at what age for the first time?** _______________Years

**H6.1 How do you feel when your child is out alone with friends?**

| | 1 | 2 | 3 | 4 | 5 | 6 | 7 | |
|---|---|---|---|---|---|---|---|---|
| I am not afraid when my child is out and about alone. | | | | | | | | I am very scared when my child is out and about alone. |

☐ Not yet applicable (toddler, young schoolchild)

**H6.2 What might you be afraid of when your child is traveling alone (without parents)?**

| | 1 | 2 | 3 | 4 | 5 | 6 | 7 | |
|---|---|---|---|---|---|---|---|---|
| Don't worry about sticking to the diet | | | | | | | | Fear that the diet will not be adhered to |
| Don't worry about not keeping to the maximum fasting time. | | | | | | | | Fear that the maximum fasting time will not be adhered to. |
| No fear of a sudden metabolic derailment | | | | | | | | Fear of a sudden metabolic derailment |

☐ Not yet applicable (toddler, young schoolchild)

**I - SPORT/ PHYSICAL ACTIVITY**

**I1. Due to his metabolic disorder, my child's physical performance is ...**

| | 1 | 2 | 3 | 4 | 5 | 6 | 7 | |
|---|---|---|---|---|---|---|---|---|
| not restricted at all. | | | | | | | | Very severely restricted |

**I2. My child can/could participate in school sports?**

| | 1 | 2 | 3 | 4 | 5 | 6 | 7 | |
|---|---|---|---|---|---|---|---|---|
| My child was always able to take part in school sports. | | | | | | | | My child was never able to take part in school sports. |

☐ Not yet applicable (small child)

**I3.1 My child does sport regularly**

☐ yes

☐ no (*<u>If no, continue with question I6.1)</u>*

**I3.2 If "YES", which sports: ____________________________________________________**

**I3.3 How often: ________________________________________( e.g. daily, weekly)**

**I4 My child reports after sport...**

| | 1 | 2 | 3 | 4 | 5 | 6 | 7 | |
|---|---|---|---|---|---|---|---|---|
| never reported muscle pain. | | | | | | | | always complain of muscle pain. |

**I5. What measures do you take to ensure an adequate energy supply during sport?**

*Multiple answers possible*

☐ Carbohydrate-rich meal before exercise

☐ Taking MCT oil/powder before exercise

☐ Ingestion of maltodextrin solution during exercise

☐ Sonstiges:________________________

☐ none

**I6.1 My child has the following sports hobbies...**

| | 1 | 2 | 3 | 4 | 5 | 6 | 7 | |
|---|---|---|---|---|---|---|---|---|
| No restrictions due to reduced load capacity. | | | | | | | | severe restrictions due to reduced resilience. |

☐ Not yet applicable (infant/toddler)

**I6.2 My child can enjoy certain sporting hobbies ...**

| | 1 | 2 | 3 | 4 | 5 | 6 | 7 | |
|---|---|---|---|---|---|---|---|---|
| because of physical complaints. | | | | | | | | because there are no physical complaints. |

☐ Not yet applicable (infant/toddler)

**I6.3 I have the impression that my child ...**

| | 1 | 2 | 3 | 4 | 5 | 6 | 7 | |
|---|---|---|---|---|---|---|---|---|
| does not suffer from its limited capacity. | | | | | | | | suffers greatly from his limited performance. |

☐ Not yet applicable (infant/toddler)

**I7.1 My child (also) pursues other non-sporting hobbies.**

☐ yes

☐ **no**

**I7.2 If "YES", which hobbies? ____________________________________________________**

ID-Nr.____________________

## K - TRAVEL

**K1. How does your child's illness make you feel about traveling?**

| | 1 | 2 | 3 | 4 | 5 | 6 | 7 | |
|---|---|---|---|---|---|---|---|---|
| I don't feel restricted at all. | | | | | | | | I feel very restricted. |

**K2. Which statements apply to you?**
*Multiple answers possible*

☐ We have not traveled as a family/with a child since my child was diagnosed.
☐ We travel within Germany.
☐ We travel within Europe.
☐ We travel outside Europe to industrialized countries (e.g. USA).
☐ We travel to developing countries outside Europe.

**K3. How much do you agree with the following statements?**

| | 1 | 2 | 3 | 4 | 5 | 6 | 7 | |
|---|---|---|---|---|---|---|---|---|
| I don't like traveling with my child because of the time-consuming planning involved. | | | | | | | | I enjoy traveling with my child despite the time-consuming planning required. |
| Traveling is always associated with particular stress. | | | | | | | | Traveling does not involve any particular stress. |
| I am afraid of traveling with my child. | | | | | | | | I'm not afraid of traveling with my child. |
| I feel insecure before traveling with my child. | | | | | | | | I don't feel unsafe before traveling with my child. |
| I am afraid of traveling to other European countries because of my child's metabolic disorder. | | | | | | | | I am not afraid of traveling to other European countries because of my child's metabolic disorder. |

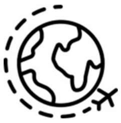

| | | | | | | | | |
|---|---|---|---|---|---|---|---|---|
| I am afraid of traveling outside Europe because of my child's metabolic disorder. | | | | | | | | I am not afraid of traveling outside Europe because of my child's metabolic disorder. |
| | **1** | **2** | **3** | **4** | **5** | **6** | **7** | |
| I am afraid of traveling to developing countries because of my child's metabolic disorder. | | | | | | | | I am not afraid of traveling to developing countries because of my child's metabolic disorder. |
| A hotel vacation with meals is difficult for us due to our child's special diet. | | | | | | | | A hotel vacation with meals is no problem for us due to our child's special diet. |

**K4.1 Does your child have an emergency protocol?**

☐ yes ☐ no

**K4.2 If "YES", in which languages do you have an emergency protocol?**

*(multiple answers possible)*

☐ In German ☐ In English

☐ Sonstige Sprachen:_______________________________________________

**K5.1 Do you or your child always carry this emergency protocol with you (when traveling)?**

☐ yes ☐ no

**K5.2 If "NO", why not?**

_______________________________________________

ID-Nr.__________________

## L- SOCIAL SUPPORT

**L.1.1 Which of the following options for state support are you aware of?** *(multiple answers possible)*

☐ Pension office (e.g. severely disabled person's pass)

☐ Youth welfare office/integration assistance (e.g. integration status)

☐ Care insurance fund (e.g. care degree)

☐ Health insurance (e.g. prescribable foods such as MCT oil)

☐ Pension fund (e.g. child rehabilitation)

**L.1.2 Where do you get information about state support options?**
*(multiple answers possible)*

☐ Social worker at the metabolic outpatient clinic/SPZ

☐ Pediatrician/family doctor

☐ Other affected families

☐ Self-help group Fett-SOS e.V.

☐ Youth welfare office/integration assistance

☐ other: __________________________________________________

**L3.1 Does my child currently have a degree of disability/severe disability certificate and, if applicable, a disability mark?**

☐ yes ☐ no

**L3.2 If "YES", how high is the recognized GdB (degree of disability)?** ___________

☐ Flag H ☐ Mark G/aG ☐ Mark B

**L4.1 My child currently has a care degree**

☐ yes ☐ no

**L4.2 If "YES", what is the current care level?** ______________

**L.5 Please mark in the following table how you feel supported by the state institutions?**

| | | | | | | | | |
|---|---|---|---|---|---|---|---|---|
| I feel very well supported | **1** | **2** | **3** | **4** | **5** | **6** | **7** | I don't feel supported at all |

| | | | | | | | | |
|---|---|---|---|---|---|---|---|---|
| **L5.1 - the pension office (issues the severely disabled person's pass)** | | | | | | | | |
| **L5.2 - the health insurance fund** | | | | | | | | |
| **L5.3 - Pension insurance** | | | | | | | | |
| **L5.4 - Long-term care insurance** | | | | | | | | |

**L5.5 Where do you need or would like more support?**

______________________________________________

______________________________________________

______________________________________________

**L6.1 Do you have a social network (family, friends) for support?**

| | 1 | 2 | 3 | 4 | 5 | 6 | 7 | |
|---|---|---|---|---|---|---|---|---|
| I have a well-functioning social network for support. | | | | | | | | I don't have a functioning social network for support. |

**L6.2 In everyday life I experience/experienced personal and organizational support from** *(multiple answers possible)*

☐ Partner

☐ other parent

☐ Grandparents

☐ Friends

☐ a care service

☐ a family helper

☐ sonstige______________________

**L6.3 I feel comfortable with my child's metabolic disorder in everyday life ...**

| | 1 | 2 | 3 | 4 | 5 | 6 | 7 | |
|---|---|---|---|---|---|---|---|---|
| never left alone. | | | | | | | | always left alone |

**L7.1 Where does your psychological support come from?**
*(multiple answers possible)*

☐ Spouse/life partner

☐ Family

☐ Children

☐ Religion

☐ Other parents/self-help group

☐ Friends

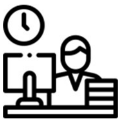

## M- EFFECTS ON PARENTS' EVERYDAY WORKING LIFE

**M1.1 What is your current occupation as a parent?**

*(multiple answers possible)*

☐ occupation/gainful employment

☐ without employment

☐ on parental leave, but previously employed

☐ on parental leave, not previously employed

☐ Study

☐ Vocational training

☐ Other: _________________________

**M1.2 If you are employed, what percentage of your time is spent working?**

*(multiple answers possible)*

☐ currently full-time (> 35h/wk)

☐ Part-time________________%

☐ I was also able to work during my child's infancy (up to the age of 3)

☐ I have suspended in the meantime (age of the child ______-______ years).

☐ I have worked part-time in the meantime (age of the child ______-______ years).

**M1.3 If you are employed, in what profession?**

______________________________________________________________________

**M1.4 Does your employer know about your child's metabolic disorder?**

☐ yes

☐ no

☐ not applicable

**M2.1 Do you feel or did you feel that your child's illness ...**

| | 1 | 2 | 3 | 4 | 5 | 6 | 7 | |
|---|---|---|---|---|---|---|---|---|
| not professionally restricted at all. | | | | | | | | severely restricted professionally. |

ID-Nr.___________________

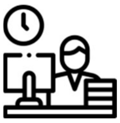

**M2.2 How much do you agree with the following statements regarding your child's metabolic disorder?**

| | 1 | 2 | 3 | 4 | 5 | 6 | 7 | |
|---|---|---|---|---|---|---|---|---|
| I have a lot of absences due to my child being hospitalized. | | | | | | | | I have no absences due to my child being hospitalized. |
| I would like to work more, but this is not possible due to my child's metabolic disorder. | | | | | | | | I would like to work less because of my child's metabolic disorder. |
| I have problems with my employer because of my child's metabolic disorder. | | | | | | | | I have no problems with my employer because of my child's metabolic disorder. |
| I would like to work less and be able to look after my child more. | | | | | | | | My workload is just right and I can take sufficient care of my child. |
| I have professional disadvantages due to my child's metabolic disorder. | | | | | | | | I have no professional disadvantages due to my child's metabolic disorder. |
| I can/was able to realize my full potential professionally, despite my child's metabolic disorder. | | | | | | | | I cannot/ could not realize myself professionally due to my child's metabolic disorder. |

**M3.1 How did having a child with a metabolic disorder affect your career decision?**

☐ It had no effect.

☐ I chose a less demanding job.

☐ I chose a job that didn't require me to travel for several days.

☐ I have given up my job.

☐ others:_____________________________

**M4.1 How often have you been absent from work (caring for your child at home or accompanying them to hospital) in the last 3 years due to your child's metabolic disorder?**

Average days/year _______________

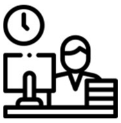

**M4.2 How often have you been ill yourself in the last 3 years (including e.g. psychosomatic exhaustion)?**

Average days/year _______________

**M4.3 How exhausted do you currently feel?**

| | 1 | 2 | 3 | 4 | 5 | 6 | 7 | |
|---|---|---|---|---|---|---|---|---|
| not exhausted at all | | | | | | | | very exhausted |

**M4.4 Have you yourself taken advantage of a rehabilitation service (rehab, mother/father/child cure) in the last 10 years (excluding accompanying person for child rehab)?**

☐ yes how often? ________________

☐ nowhy not?___________________

## N- EFFECTS ON FAMILY LIFE

**N1.1 To what extent has having a child with a metabolic disorder affected your relationship with your partner?**

*(multiple answers possible)*

☐ has brought us closer together

☐ Has made us better communication partners

☐ Causes conflicts

☐ caused us to part ways

☐ No significant influence

**N2. I have the feeling that my child's metabolic disorder is a problem for unaffected siblings ....**

| | 1 | 2 | 3 | 4 | 5 | 6 | 7 | |
|---|---|---|---|---|---|---|---|---|
| is not a burden. | | | | | | | | represents a major burden. |

☐ no siblings

**N4.1 The relationship...?**

| | 1 | 2 | 3 | 4 | 5 | 6 | 7 | |
|---|---|---|---|---|---|---|---|---|
| is always interested in my child's metabolic disorder. | | | | | | | | is never interested in my child's metabolic disorder. |
| understands my child's metabolic disorder. | | | | | | | | has no understanding of my child's metabolic disorder. |
| always trivializes and plays down my child's metabolic disorder. | | | | | | | | never trivializes or plays down my child's metabolic disease. |

**N5.1 Perceived financial burden**

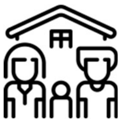

| | 1 | 2 | 3 | 4 | 5 | 6 | 7 | |
|---|---|---|---|---|---|---|---|---|
| The metabolic disorder is not a financial burden. | | | | | | | | The metabolic disorder represents a major financial burden. |

**N5.2 Additional monthly financial burden due to the metabolic disorder (e.g. vitamin supplements, fish oil, aids, therapies, visits to the doctor, self-help meetings, ...)**

☐ < 50€

☐ 51- 100€

☐ 101 - 200€

☐ 201 - 500€

☐ 501 - 1000€

☐ > 1000€

## O- GENERAL

**O1.1 How often do you currently think about what consequences the metabolic disorder will have on your child's future life?**

☐ several times a day

☐ daily

☐ at least once a week

☐ once a month or less often

☐ only when I have an appointment at the metabolic outpatient clinic

☐ others:_______________________________________________

**O1.2 How often do you think about the possibility of your child's death?**

☐ daily

☐ several times a week

☐ once a week

☐ irregular

**O2. On the scale below, please place a cross where your personal opinion on the following two statements lies:**

| | 1 | 2 | 3 | 4 | 5 | 6 | 7 | |
|---|---|---|---|---|---|---|---|---|
| My child's metabolic disorder is **not a** serious illness. | | | | | | | | My child's metabolic disorder is **a** serious illness. |
| I find it difficult to treat the metabolic disorder. | | | | | | | | Treating the metabolic disorder is not difficult for me. |
| If you pay attention to a few special features, you can lead a normal life even with a congenital fatty acid oxidation disorder. | | | | | | | | If you have a congenital fatty acid oxidation disorder, you are ill and cannot lead a normal life. |

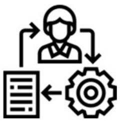

## P- EFFECTS OF YOUR CHILD'S METABOLIC DISORDER ON HIS OR HER LIFE

**P1. How do you assess the impact of your child's metabolic disorder on various areas of your child's life (including future life)?**

| No effects | 1 | 2 | 3 | 4 | 5 | 6 | 7 | Very large effects |
|---|---|---|---|---|---|---|---|---|
| Physical performance | | | | | | | | |
| Leisure activities | | | | | | | | |
| Friendships | | | | | | | | |
| Partnership | | | | | | | | |
| Economic effects (e.g. treatment costs, influences on education and vocational training, career choice and employment) | | | | | | | | |
| Sick leave at the place of training or work | | | | | | | | |
| Intellectual performance | | | | | | | | |
| Emotional situation | | | | | | | | |

**P2. How often do you experience the following feelings in connection with your child's metabolic disorder?**

| I never have... | 1 | 2 | 3 | 4 | 5 | 6 | 7 | I have very often... |
|---|---|---|---|---|---|---|---|---|
| Joy | | | | | | | | |
| Anger | | | | | | | | |
| Fear | | | | | | | | |
| Disgust | | | | | | | | |
| Mourning | | | | | | | | |
| Fear | | | | | | | | |
| Shame | | | | | | | | |
| Serenity | | | | | | | | |
| Despair | | | | | | | | |
| Guilt | | | | | | | | |
| Embarrassment | | | | | | | | |
| Trouble | | | | | | | | |
| Surprise | | | | | | | | |

**P3.1 What stresses has a child with a metabolic disorder brought into her life?**
*(multiple answers possible)*

☐ Financial

☐ Loss of friends

☐ Emotional

☐ Mental

☐ Interpersonal matters.

☐ Lack of free space

☐ Feeling of isolation

☐ Uniqueness

**P3.2 How has the presence of a child with a metabolic disorder changed their daily activities?**

*(multiple answers possible)*

☐ Fewer social contacts.

☐ Less personal time for me.

☐ Less time for the other (sibling) children.

☐ More time for my children.

**P3.3 Having a child with a metabolic disorder had the following positive effects:** *(multiple answers possible)*

☐ Made me more compassionate.

☐ turned me into an activist.

☐ showed me my strengths.

☐ Made me more patient.

☐ Has led to a change in my world view.

☐ others___________________________________.

**P3.4 How has having a child with a metabolic disorder affected your family planning?**

☐ We decided to have more children despite the risk of the metabolic disorder.

☐ Decision to have more children, regardless of the metabolic disorder.

☐ No decision yet on further family planning.

☐ We hadn't planned to have more children.

**P.3.5 Have you ever received genetic counseling regarding the metabolic disorder?**

☐ yes ☐ no

**P4.1 Communication regarding the disease:**
**The following persons are informed about my child's illness**
*(multiple answers possible)*

| | YES | NO | Not applicable |
|---|---|---|---|
| Other parent/partner | | | |
| Older siblings | | | |
| Younger siblings | | | |
| Family members (except parents/siblings) | | | |
| Friends of the parents | | | |
| Friends of the child | | | |
| Sports mates of the child | | | |
| Educator/ Teacher | | | |
| Colleagues | | | |
| Supervisor | | | |
| other _______________________________________________ | | | |

**P4.2 I deal with my child's illness ...**

| | 1 | 2 | 3 | 4 | 5 | 6 | 7 | |
|---|---|---|---|---|---|---|---|---|
| openly. | | | | | | | | not openly. |

**P4.3 About my child's metabolic disorder...**

| | 1 | 2 | 3 | 4 | 5 | 6 | 7 | |
|---|---|---|---|---|---|---|---|---|
| nobody should know. | | | | | | | | everyone can know about it. |

**P5.1 Are you a member of the self-help group for congenital fatty acid oxidation disorders (Fett-SOS e.V.)?**

☐ yes ☐ no

**P5.2 If "NO", why not?** ____________________________________________

**P5.3 If "YES", how do you feel about the exchange with other affected families?**

| | 1 | 2 | 3 | 4 | 5 | 6 | 7 | |
|---|---|---|---|---|---|---|---|---|
| not helpful at all | | | | | | | | very helpful |
| Not at all supportive | | | | | | | | very supportive |
| exhausting | | | | | | | | energy-saving |

**P6.1 Is your child in psychological care?**

☐ Permanent ☐ Temporary ☐ no

**P6.2.Are you under psychological care?**

☐ Permanent ☐ Temporary ☐ no

**P6.3 Psychological support...**

*(multiple answers possible)*

☐ should be offered to all affected families.
☐ should be offered to both patients and their parents.
☐ should also be offered to siblings.
☐ is only necessary in the first few weeks.

**Is there anything else you would like to tell us?**

Thank you very much!
